# Power and entitlement: Addressing the structural drivers of food insecurity and malnutrition

**DOI:** 10.1371/journal.pgph.0007192

**Published:** 2026-09-01

**Authors:** Helen Walls, Martin McKee

**Affiliations:** Faculty of Public Health & Policy, London School of Hygiene & Tropical Medicine, United Kingdom; Brigham and Women’s Hospital, UNITED STATES OF AMERICA

## Abstract

The world produces enough food for all, yet it falls short of providing a nutritionally balanced diet, and what it produces is extremely unevenly distributed, with high levels of undernourishment and obesity co-existing, often within the same countries. Policy responses have achieved only limited success because they often fail to address the underlying causes of these outcomes. Drawing on Amartya Sen’s entitlement framework and adapting it by making the conditions governing conversion analytically explicit, we explain food access in terms of people’s endowments and the exchange, production, and transfer entitlements through which food is acquired. We examine two analytically distinct but mutually reinforcing forces: corporate power and wealth inequality. Corporate concentration can reshape production, supply chains, food environments and public policy, while wealth inequality limits access to productive assets, purchasing power and social protection. We argue that durable progress requires policies that rebuild household endowments, constrain excessive corporate and political power, strengthen conversion factors, and widen exchange, production and transfer entitlements. Priority measures include progressive taxation of income and particularly taxation of extreme wealth, social protection, competition and governance reform, secure land tenure and support for smallholders, regulation of food marketing, and investment in enabling infrastructure.

## Introduction

The world produces enough food to meet the caloric needs of everyone on the planet, though not to ensure a nutritionally balanced diet for all [1,2]. The food that is produced and consumed is, however, extremely unevenly distributed, with high levels of undernourishment and food insecurity co-existing with obesity, often within the same countries [3]. In 2023, an estimated 733 million people worldwide, equivalent to approximately one in eleven people globally, and up to one in five in Africa, were undernourished, while over three billion people could not afford a healthy diet [4,5]. Women and girls are disproportionately affected, comprising nearly 60% of the world’s hungry population [6]. Meanwhile, 38% of the world’s population is overweight or obese [7]. These coexisting forms of malnutrition make frameworks explaining who can obtain nutritious food and under what conditions essential to effective policy.

An extensive body of literature has documented this maldistribution of food and, although policy interventions designed to redress it, including nutrition programmes, food assistance, market-based measures and obesity-prevention policies, have sometimes led to meaningful, if uneven, improvements, they have failed to provide lasting change [8–11]. Many programmes address immediate shortfalls or individual behaviour but leave intact the distribution of assets, market power and political influence that shapes food access. They can therefore mitigate entitlement failure without changing the structural conditions that reproduce it.

We use the widely adopted definition developed by the Food and Agriculture Organization, under which food security is understood as a condition in which all people, at all times, have physical, social, and economic access to sufficient, safe, and nutritious food that meets dietary needs for an active and healthy life [12]. This definition encompasses the availability of food, access to it, its nutritional quality, stability over time, and the sustainability of food systems. Achieving food security requires coordinated conditions across the entire food chain, from production and distribution to consumption. However, understanding why the international community has failed to address the fundamental reasons food is unevenly distributed requires examining the structures and processes that determine who holds power within the global food system and what shapes access to food.

Two analytically distinct but mutually reinforcing issues stand out. The first is the concentration of power in large corporations relative to individual consumers, workers and small producers. This power is structural, arising from firms’ positions in markets and supply chains; instrumental, exercised through lobbying and policy processes; and ideational, exercised by shaping knowledge, norms and preferences. A small number of multinational food and beverage corporations dominate important parts of global food systems [13]. Concentration varies by sector, so no single global market-share figure is meaningful. By 2020, however, four firms controlled approximately 50–60% of the global commercial seed market and 60–70% of the global pesticide market [13]. Major corporations such as Nestlé, PepsiCo, and Cargill generate annual revenues comparable to, or exceeding, the GDP of many mid-sized national economies. Their influence contributes to the widespread provision of low-cost, energy-dense, nutrient-poor products that alleviate hunger but fail to nourish [14]. Together with similarly powerful corporations in the agrichemical and retail sectors, they entrench an economic model that prioritises profit maximisation over human well-being.

The second, less well-recognised but critically important issue is extreme wealth inequality. A large body of evidence demonstrates how current economic models condemn many households to precarious living conditions, characterised by uncertain employment, unstable incomes, and limited access to essential goods, including food [15], with profound consequences for health across the life course [16]. This economic precarity itself increases the cost of living for households: those reliant on private rental markets often face higher housing costs, while limited access to transport or digital infrastructure restricts their ability to secure affordable food and other necessities [15]. In more severe cases, these pressures lead to individuals falling into debt spirals that are nearly impossible to escape.

The scale of contemporary wealth inequality is striking. Between 2020 and 2023, the wealthiest 1% of the population captured nearly two-thirds of all newly generated wealth, approximately US$ 42 trillion, a figure almost twice the amount accrued by everyone in the bottom 99% combined [17]. At the end of the distribution, one of the world’s billionaires (unnamed) gained US$ 1.7 million for every US$ 1 of new global wealth earned by the average person in the bottom 90%.

This gap continues to widen. In 2024 alone, global billionaire wealth increased by $2 trillion, three times faster than in 2023, at a time when 44% of the world’s population lived in poverty as defined by the World Bank [18]. In 2025, billionaire wealth rose by more than 16%, three times the average annual increase over the previous five years. The world’s 12 richest billionaires now hold more wealth than the poorest half of humanity (four billion people) [19].

Corporate profitability is one mechanism linking corporate concentration to wider wealth inequality. In 2022, 95 food and energy corporations more than doubled their profits, amassing $306 billion in windfall earnings, 84% of which were distributed to shareholders [6]. The point is not that all corporations or shareholders benefit equally, but that exceptional profits in highly concentrated sectors can be transferred predominantly to asset owners, reinforcing the concentration of wealth.

These dynamics are pushing more of the world’s ‘middle classes’ into economic precarity. In the UK, the Food Foundation reported in 2024 that 15% of UK households had recently gone hungry or skipped meals, rising to one in five families with children [20]. A separate study found that 20% of those in the middle fifth of the UK’s income distribution struggled to afford food and other essentials [21].

Despite this evidence, the relationship between widening wealth inequality and growing food insecurity remains poorly integrated with work on corporate power. Research on food sovereignty and the political economy and commercial determinants of food systems has examined concentrated corporate influence, while scholarship on inequality has documented unequal access to land, labour, income and food [13,22–24]. Less attention has been given to bringing these literatures together within a common framework that traces how both forces shape household food access. This paper addresses that gap by synthesising a broad, interdisciplinary body of literature.

While prior work has addressed corporate power and wealth inequality individually, the novel contribution of this paper is to conceptualise them as analytically distinct but mutually reinforcing entitlement-shaping forces within Amartya Sen’s framework. We first examine the mechanisms associated with each separately and then show how they can jointly restructure endowments, conversion factors, and exchange, production and transfer entitlements. In this framework, endowments are the resources people possess, conversion factors determine how effectively those resources can be used, and entitlements are the recognised means through which food is acquired. This framing helps explain why food insecurity persists despite near-sufficient production and trade.

### Entitlements

Food production, food availability, and food access are distinct yet interconnected concepts [25]. Food production and availability are determined primarily by market forces and are subject to constraints such as climate, infrastructure, transport, and trade regimes. Production refers to the quantity of food produced, while availability in a given location reflects domestic production minus exports plus imports. Food access is more complex, as it concerns how individuals and households obtain food. We propose that food access, and its relationship to power and wealth inequality, can be understood through Sen’s theory of “entitlements”.

In his seminal work Poverty and Famines (1981), Nobel Laureate Amartya Sen [26] observed that famines occur even when food is available in affected areas. The tragedies arose not from a lack of food, but from failures in people’s ability to obtain it for consumption, what Sen termed entitlement failure. We use Sen’s entitlement framework in an adapted form (Table 1). Sen’s original formulation centres on endowments and entitlement relations; the paper makes the social, political and economic conditions governing their conversion into food analytically explicit as ‘conversion factors’ [26,27].

**Table 1:** Summary of Sen’s entitlement framework.

Component	Meaning	Food-access relevance	Examples
Endowments	Resources held by individuals or households.	The resource base from which food can be produced or obtained.	Land, livestock, labour, income, education, assets and social capital.
Conversion factors	Conditions determining how effectively endowments can be used.	Determine whether resources can be translated into production, purchasing power or support.	Tenure rights, markets, transport, credit, non-discrimination, political rights and regulation.
Exchange entitlements	Food obtained through buying, selling or trading.	Depend on purchasing power and food-market structure.	Wages, prices, employment, retail access and information.
Production entitlements	Food produced directly using one’s resources.	Depend on productive assets and the capacity to use them.	Farming, gardening, livestock and wild-food harvesting.
Transfer entitlements	Food or resources obtained through transfers.	Protect when production or exchange entitlements are inadequate.	Social protection, food assistance, remittances, family support and gifts.

The first element, endowments, refers to the resources that individuals or households possess, such as land, livestock, labour, education, and social capital. Empirical studies illustrate the importance of endowments for food security. For instance, in South India, secure tenure, especially when formally recognised, has been associated with greater food security [28]. Similar findings have been documented in Nigeria [29] and Uganda [30], where secure land tenure has also been linked to increased dietary diversity. Evidence from Ethiopia further shows that livestock ownership is positively associated with food security outcomes [31].

The second element, conversion factors, determines the extent to which endowments can be transformed into effective access to food. Social, political, and economic conditions influence these factors. For example, land ownership is insufficient to achieve food security if individuals and households lack legal rights, market access, or the means to cultivate or sell land productively. Similarly, discrimination in labour markets can reduce the returns on education, weakening its protective effect on food security [32]. Political rights, including the ability to participate in political processes, also function as conversion factors by conferring influence over policies that shape food systems, and have been associated with improved food security outcomes [33,34].

The third element, entitlements, refers to the specific ways in which food is acquired. Sen distinguishes between exchange entitlements, production entitlements, and transfer entitlements, each corresponding to a different mode of food access. These modes operate within what has been described as the “food environment”, the interface or ‘link’ through which people interact with the wider food system [35]. Sources of food in local food environments include supermarkets, informal markets, own-farm production, wild food harvesting, and social transfers such as gifts or state transfers. Their characteristics, such as the types of vendors present, the range, quality, and processing of foods available, pricing structures, and the information provided to consumers, shape and constrain food choices.

Exchange entitlements refer to the food that individuals or households can obtain through market transactions. They are shaped by what people can buy, sell or trade, based on their endowments, such as income, assets and labour power, and on conversion factors that determine how effectively these endowments can be translated into purchasing power, including prices, employment security, access to credit, transport, information, and market regulation. These entitlements are realised within local food environments, where market structures, such as the dominance of supermarkets, availability of informal traders, and pricing and product strategies, determine what food is available, affordable, and accessible.

Production entitlements arise from the ability to produce food directly from one’s own resources, such as through farming, gardening or livestock rearing. These entitlements depend on individual and household endowments and the conversion factors that enable productive use, and are shaped by local agroecological and institutional contexts.

Transfer entitlements refer to access to food obtained through transfers from others, including formal mechanisms such as government social protection programmes and informal channels such as family support, community networks, remittances, and gifts. The reliability of these entitlements depends on an individual or household’s social endowments and on institutional arrangements that facilitate or constrain redistribution.

Taken together, this adaptation of Sen’s entitlement framework provides a way to understand food access as the product of interacting endowments, conversion conditions and entitlement relations. In the sections that follow, we apply this framework to examine how corporate concentration and extreme wealth inequality reshape food access. Specifically, we analyse how these forces affect: (i) individual or household endowments; (ii) the conversion of endowments into food through social, political, and market processes; and (iii) exchange, production and transfer entitlements within local food environments.

### How does corporate power affect entitlements?

Corporate concentration affects food access by reshaping all three components of Sen’s entitlement framework: it alters individual and household endowments, constrains conversion factors, and narrows exchange, production and transfer entitlements.

Although the size and scope of commercial entities vary significantly, the largest multinational and transnational corporations exert disproportionate influence over food systems [36] and play a critical role in shaping the structures and processes associated with entitlement theory. First, they influence agricultural practices; for example, by promoting monocultures, these corporations reshape agricultural production. Where genetically modified crops are permitted, they can control the intellectual property associated with crop genomes and, in some cases, the pesticides or herbicides required to cultivate them [37,38]. Through direct control or strategic partnerships with agrochemical companies, they limit farmers’ autonomy, prioritising high-yield, commercially profitable crops over diverse and sustainable farming practices. This shifts production away from own-farm systems toward market-oriented models and, from an entitlement perspective, primarily weakens production entitlements by limiting farmers’ control over what and how they produce.

Second, large corporations dominate much of the food supply chain, from processing raw agricultural products to their global distribution [13]. This centralised model favours large-scale operations while marginalising smallholders and local or regional food economies [36,39]. Concentrated firms can shape contractual terms, quality standards, input packages, purchasing conditions and access to logistics and storage, while their capacity to redirect sourcing gives them substantial bargaining power [40]. Smaller countries and producers may consequently become dependent on corporate-controlled supply chains [41]. Farmers can face lower or more volatile farm-gate prices, higher input costs, stringent standards without corresponding support, and exclusion from distribution networks. These dynamics constrain conversion factors by weakening farmers’ ability to translate land, labour and skills into reliable market access, income and secure food.

Third, global corporations shape local food environments and consumer preferences through aggressive marketing and branding strategies that promote highly processed and convenience foods high in sugar, salt, and unhealthy fats [42,43]. Much of the information about product quality and safety that people now receive within local food environments is increasingly mediated by these promotional practices, which operate across multiple channels, using increasingly sophisticated methods such as experience-based marketing, targeted advertising using personal data, payments to influencers, and product placements in digital games [35,44]. Vulnerable populations with already limited entitlements may be targeted, thereby increasing reliance on foods incompatible with traditional or healthy diets. These dynamics narrow exchange entitlements by shaping what foods are available, affordable and socially acceptable.

Finally, large corporations exert significant political influence in ways that are often legal but ethically dubious. This includes funding biased research to support lobbying aimed at delaying regulation, securing favourable trade agreements or obtaining subsidies, while also seeking to capture regulatory processes [45–47]. Agricultural subsidies, intellectual property rules, and land-use policies can promote proprietary inputs and large-scale production, thereby weakening smallholders’ production entitlements. Competition, trade, pricing and marketing rules shape which foods are available and affordable, affecting exchange entitlements. Taxation, public expenditure and eligibility rules influence food assistance and social protection, affecting transfer entitlements. Across all three, lobbying and regulatory capture alter conversion factors by changing the legal and institutional conditions under which households turn resources into food.

Taken together, these mechanisms illustrate that food entitlements are not simply determined by individual resources or market participation, but are structurally shaped by corporate power. By influencing what is produced, how it is distributed, what is promoted, and how regulation is designed, large corporations reconfigure the institutional and economic conditions under which people convert resources into food. In this way, corporate concentration does not merely constrain individual or household food choices; it reshapes the entitlement system itself, embedding asymmetries of power into the processes of production, exchange, and transfer across food systems.

### How do wealth inequalities affect entitlements?

Wealth inequality affects food access not only through income shortages but also by reshaping the entitlement structure itself. In this section, we show how inequality: erodes individual and household endowments; limits the conversion of resources into food; and narrows exchange, production and transfer entitlements across societies.

At one level, the relationship is straightforward: unequal access to resources limits poorer individuals’ and households’ ability to afford food or obtain it at reasonable prices. A review by Shostak demonstrates how inequality shapes access to land, labour, and food in ways that disproportionately disadvantage marginalised communities, particularly along lines of race, class, and gender [24]. Wealth inequality erodes exchange entitlements by limiting the ability to translate income, labour and assets into food. It also weakens production entitlements when land and other productive assets become concentrated, smallholders are displaced or compelled to sell, and households lose direct access to food production. This echoes the process described by Polanyi, in which the commodification of land and labour can produce social dislocation and deprivation [48].

However, in contexts of increasing wealth inequality, the erosion of exchange entitlements extends well beyond those in poverty. Even those with financial resources may struggle over time to convert their endowments into secure access to food through markets, thereby expressing exchange entitlements. Wealth exists along a continuum, and its societal distribution shapes whose preferences count in markets and whose needs remain unmet (Table 2).

**Table 2:** Structural drivers, entitlement effects and corresponding policy responses.

Primary force	Effects on endowments	Effects on conversion factors and entitlements	Policy responses
Corporate concentration and power	Can reduce farmer autonomy, labour bargaining power and control over productive assets, amongst other impacts.	Production: proprietary inputs and sourcing can constrain production. Exchange: supply chains, pricing, product mixes and marketing shape affordability and choice. Transfer: political influence can affect social provision.	Competition policy; supply chain and lobbying transparency; marketing regulation; public procurement; secure tenure; support for smallholders, cooperatives, storage, and extension.
Extreme wealth inequality and financialisation	Concentrates land, housing and financial assets; increases debt; weakens labour-based endowments.	Production: land concentration can displace smallholders. Exchange: unequal purchasing power and high essential costs reduce access. Transfer: elite influence can weaken redistribution.	Progressive income taxation and taxation of extreme wealth; fair wages and collective bargaining; asset-building and land reform; social protection; rights-based policies; enabling infrastructure.
Mutually reinforcing effects	Corporate distributions can intensify wealth concentration, while wealth increases political and market influence.	Cumulative effects can simultaneously narrow several entitlements and undermine democratic accountability.	Combine immediate protection with structural reforms that redistribute resources and political power.

First, market structures are disproportionately shaped by those with the greatest purchasing power. Individuals and households able to convert their needs into ‘effective demand’, the willingness and ability to purchase goods at prevailing prices, exert greater influence over what food is produced, distributed and made locally available. This does not mean that suppliers ignore lower-income consumers: many pursue profits through high-volume, low-margin markets, including informal and street-food markets. Rather, unequal purchasing power affects expected margins, investment and the quality and range of products supplied. Where affluent and low-income populations are geographically separated, these dynamics can contribute to food deserts [49,50]: local food environments in which fresh food is scarce or relatively expensive. These processes erode exchange entitlements by tying food availability and affordability to unequal purchasing power.

Second, these trends form part of the wider financialisation of economies, corporations and households since the 1980s. Financialisation has elevated the role of financial markets and institutional investors, strengthened shareholder primacy and encouraged firms to prioritise returns to shareholders, while exposing households more directly to debt and asset-price dynamics [51]. Wealth concentration at the top is both a driver and an outcome of these processes. Over recent decades, wealth has become increasingly concentrated among asset holders [52]. Unlike wage-dependent households, high-wealth individuals derive substantial income from capital returns. At the aggregate level, wealth has become increasingly concentrated among asset holders, while high-wealth households derive a much larger share of their income from capital than wage-dependent households do. Where returns on capital exceed wage growth, this can reinforce cumulative wealth concentration, although the proportion of capital income that is reinvested varies across households and over time.

Political-economy considerations indicate that such dynamics contribute to asset-price inflation and reinforce structural imbalances in which returns to capital outpace wage growth. As wage growth consistently lags behind returns to capital, the relative purchasing power of wage-dependent households declines. From an entitlement perspective, this weakens exchange entitlements for the majority of the population by increasing the cost of essentials, including food and housing, relative to earned income.

Third, wage stagnation and labour market precarity constrain the majority. Across many advanced economies in recent years, particularly in more neoliberal and Anglophone contexts [53–59], real wage growth has stagnated while living costs have risen. The result is a widening gap between asset-based wealth accumulation at the top and income growth for the majority. This dynamic reduces exchange entitlements not only for those below conventional poverty thresholds but also, increasingly, for middle-income households facing rising food prices, housing costs, and insecure employment. Automation and the expansion of artificial intelligence may intensify these pressures by further weakening labour’s bargaining power [60].

These trends also extend beyond markets into the political sphere. Rising wealth inequality is closely associated with increased political influence among economic elites, a pattern widely documented in comparative political-economy research on elite capture and the structural power of capital [61,62]. Oxfam’s 2026 analysis estimates that billionaires are more than 4,000 times as likely to hold political office as ordinary citizens [19], underscoring the extent to which extreme wealth translates into direct political representation and agenda-setting power. In contexts of high wealth concentration, regulatory capture, weakened taxation capacity, and reduced public accountability become more likely, particularly where campaign finance systems and concentrated media ownership amplify elite influence. Under such conditions, governments facing fiscal pressure, often intensified by crisis-related borrowing, such as during the Covid-19 pandemic, may prioritise fiscal consolidation through austerity over redistribution, leading to the retrenchment of welfare provision and social safety nets [63]. From an entitlement perspective, these dynamics directly erode transfer entitlements for ordinary people and constrain key conversion factors by reshaping the institutional and regulatory environment through which food access is mediated. In short, the distribution of societal wealth matters profoundly. As wealth becomes more concentrated, exchange entitlements are weakened not only among the poorest but increasingly across middle-income groups. Simultaneously, transfer entitlements are undermined through reduced state capacity to provide public safety nets and political resistance to redistribution. Wealth inequality thus reshapes the entitlement structure, affecting both the market and the state as mechanisms for securing food security.

### Solutions

Addressing food insecurity in a sustained manner requires policy responses that confront the structural drivers identified above. If corporate concentration and extreme wealth inequality reshape household endowments, distort conversion factors, and narrow exchange, production and transfer entitlements, then effective policy must intervene at each of these levels. While short-term mitigation remains necessary, particularly in times of crisis, durable change depends on rebalancing the distribution of economic and political power that structures food access.

The following framework organises potential interventions according to the components of Sen’s entitlement approach: (i) rebuilding individual and household entitlements; (ii) strengthening institutional and political conversion factors; and (iii) widening exchange, production and transfer entitlements. Within each domain, we distinguish between near-term and longer-term structural reforms.

### 1. Rebuilding individual and household endowments: Addressing wealth inequality

Individual and household endowments, such as income, assets, land, labour power, and social capital, form the foundation of food access. Extreme wealth inequality erodes these endowments directly by concentrating ownership of land, housing, financial assets, and productive capital among a narrow segment of the population, while increasing debt dependence and limiting intergenerational asset accumulation for others. It also erodes them indirectly by generating macroeconomic and political dynamics, such as asset price inflation, wage stagnation, and weakened labour bargaining power, that reduce the relative purchasing power and economic security of wage-dependent households. In such contexts, even rising nominal incomes may fail to strengthen exchange entitlements, as returns to capital outpace wage growth and essential costs, particularly housing, absorb a growing share of household resources. Strengthening food security, therefore, requires policies that expand and protect the resource bases of individuals and households.

### 1.1 Near-term measures

Progressive taxation of income and expanded social protection are among the most direct tools for strengthening individual and household endowments. Evidence from cash transfer programmes in low- and middle-income countries demonstrates consistent improvements in food security and dietary quality when predictable transfers are provided [64,65]. Shock-responsive safety nets, unemployment protection, and child benefits can stabilise purchasing power during economic and climatic disruptions, preventing temporary shocks from becoming chronic entitlement failures. Minimum wage protections, strengthened collective bargaining institutions, and enforcement of labour standards can also enhance labour-based endowments by improving income security and reducing precarity.

### 1.2 Structural reforms

In situations of extreme wealth inequality, reducing structural wealth concentration becomes essential. Policies aimed at more effectively taxing net wealth, inheritance, and capital gains can help slow the accumulation of assets and generate fiscal space for redistribution. However, given the scale and mobility of contemporary capital, proposals for coordinated international taxation have gained increasing political traction. Brazil’s proposal for a global 2% tax on billionaires [61], alongside advocacy from groups such as Patriotic Millionaires, Millionaires for Humanity, and Tax Me Now, reflects shifting political norms around the legitimacy of extreme wealth concentration. In 2025, the “We Must Draw The Line” letter, signed by more than 370 millionaires and billionaires from 22 countries [62,66], urged world leaders to address the social and democratic harms of excessive wealth accumulation through more progressive taxation.

At the same time, historically high returns to capital, often substantially exceeding wage growth, mean that redistribution must form part of a broader structural strategy. Over the past century, average real returns on major equity indices have consistently outpaced inflation and, in many periods, real wage growth. In such conditions, modest taxation alone is unlikely to reverse the process of cumulative concentration. Reform must therefore aim not only to raise revenue but also to slow and ultimately rebalance the mechanisms through which extreme wealth reproduces itself across generations.

Alongside taxation of extreme wealth, asset-building policies, such as expanded access to land, affordable housing, cooperative ownership models, and inclusive financial systems, can strengthen the productive and financial endowments of lower- and middle-income households. The lowering of tax on workers’ incomes may play a role here too. These measures move beyond poverty alleviation toward reshaping the underlying distribution of economic resources that determines who commands effective demand within food systems. By broadening asset ownership and moderating capital concentration, structural reforms strengthen individual and household endowments directly and alter the macroeconomic conditions that shape food entitlements over time.

### 2. Strengthening conversion factors: Institutional and political reform

Endowments alone do not guarantee food security. Their effectiveness depends on the institutional, legal, and political conditions that enable individuals and households to convert resources into food. Corporate concentration and wealth inequality distort these conversion factors by shaping regulatory environments, influencing trade rules, and weakening democratic accountability.

### 2.1 Regulatory and governance reform

Strengthening competition policy and limiting excessive market concentration are central to restoring equitable conversion conditions. Transparent supply chains, independent oversight, and protections against regulatory capture can reduce dominant firms’ ability to shape markets and policy to their advantage. Campaign finance reform, lobbying transparency, and conflict-of-interest regulations are similarly important in preventing the erosion of democratic accountability.

### 2.2 Rights-based and legal frameworks

Embedding the Right to Food within constitutional or statutory frameworks can strengthen conversion factors by creating enforceable obligations on states. When supported by effective institutions and judicial independence, rights-based approaches can enhance programme coverage, improve administrative responsiveness, and provide mechanisms for redress when entitlements are denied.

### 2.3 Public investment in enabling infrastructure

Investments in transport, storage, digital access, and local market infrastructure can enhance households’ ability to translate income or productive assets into reliable access to food. Anti-discrimination enforcement in labour and credit markets further ensures that education, land ownership, or entrepreneurial activity translates into meaningful food entitlements.

### 3. Widening exchange, production and transfer entitlements

If corporate concentration and wealth inequality narrow how people can obtain food, widening entitlements requires restructuring the institutional and market conditions that shape food access. This means expanding substantive options across the domains of exchange, production, and transfer while reducing the structural constraints embedded in contemporary food systems.

### 3.1. Expanding exchange entitlements

Exchange entitlements depend not only on income but on the structure of food markets and local food environments. Where supply chains and retail sectors are highly concentrated, dominant firms shape availability, pricing and consumer information in ways that prioritise profitability over nutrition. Strengthening exchange entitlements, therefore, requires reshaping market conditions and purchasing power.

Effective competition policy can limit excessive concentration in retail, processing, and input markets, thereby reducing dominant firms’ ability to dictate prices and product mixes. Regulation of food marketing, particularly to children, alongside mandatory front-of-package labelling and restrictions on misleading health claims, can reduce informational asymmetries that skew consumption toward ultra-processed products. Public procurement policies, including school and hospital meal programmes, can further shift demand toward nutritious foods and more diverse suppliers.

Together, these types of measures expand exchange entitlements by making healthier foods more available, affordable and desirable within local food environments.

### 3.2. Strengthening production entitlements

Production entitlements remain central in many rural and low-income contexts, yet consolidation in seed, input and commodity markets has reduced farmer autonomy and increased dependence on global supply chains [13].

Secure land tenure, support for smallholder farmers, and cooperative marketing structures can strengthen production entitlements by increasing control over productive resources and improving bargaining power. Land reform is particularly important where historical inequalities have restricted access to assets, reinforcing both endowments and direct food-producing capacity.

Public investment in agroecological practices, local storage and extension services can enhance resilience, reduce dependence on proprietary inputs, and diversify local supply. Strengthening production entitlements in this way improves food access directly while rebalancing power within food systems.

### 3.3. Expanding transfer entitlements

Transfer entitlements buffer households against market volatility, employment insecurity and environmental shocks. Yet in highly unequal societies, social protection systems are often underfunded or politically contested [67].

Universal or near-universal social protection, such as child benefits, pensions, unemployment insurance and food assistance, can stabilise food access across the life course and reduce the risk that temporary shocks become chronic deprivation. In contexts of automation and climate disruption, proposals such as Universal Basic Income seek to secure baseline transfer entitlements independent of labour market volatility [68,69]. If financed progressively, such schemes could simultaneously strengthen household security and moderate the dynamics of wealth concentration.

Expanding transfer entitlements, therefore, both prevents immediate entitlement failure and reinforces the redistributive foundations of a more equitable food system.

## Conclusion

Policies aimed at increasing food production or improving the short-term affordability of food, while valuable, cannot on their own resolve persistent food insecurity. Sen’s entitlement framework shows why: food access depends on the resources people possess, the institutional conditions that allow those resources to be converted into food, and the exchange, production and transfer entitlements available to them. Corporate concentration and wealth inequality are analytically distinct, but they can reinforce one another by concentrating assets and decision-making power, weakening farmers’ and workers’ bargaining positions, shaping food environments, and limiting the fiscal and political space for redistribution. Durable food security therefore requires action across the entitlement system: rebuilding individual and household endowments through fair wages, progressive income taxation and particularly taxation of extreme wealth, asset-building and social protection; strengthening conversion factors through competition policy, transparent governance, rights-based frameworks and public infrastructure; and widening entitlements through healthier food markets, secure land tenure and support for smallholders, and reliable social provision. Food insecurity is not simply a problem of insufficient supply or poor individual choice. It is a distributional and political problem. By explicitly targeting wealth concentration and corporate power, policy can move beyond repeated crisis response and limited impact toward more equitable, accountable and resilient food systems.
